# A qualitative study exploring teachers’ beliefs regarding breastfeeding education in family and consumer sciences classrooms

**DOI:** 10.1186/s13006-022-00510-8

**Published:** 2022-09-21

**Authors:** Nicola Singletary, Jackie Bruce, L. Suzanne Goodell, April Fogleman

**Affiliations:** North Carolina State University, Raleigh, NC USA

**Keywords:** Breastfeeding, Infant feeding, Education, School, Family and consumer sciences

## Abstract

**Background:**

Research shows that elementary and secondary school children have considered infant feeding choices for when they become parents and are interested in learning about breastfeeding in school. Despite recommendations to include infant feeding education in secondary school classrooms, teachers’ practices and attitudes regarding this topic have been the subject of minimal research. The purpose of this study was to explore North Carolina, USA, family and consumer sciences teachers’ infant feeding education practices and their views on incorporating breastfeeding education in the curricula of family and consumer sciences classes that cover topics related to parenting and nutrition.

**Methods:**

The study used a purposive sample of 19 teachers who participated in semi-structured qualitative telephone interviews exploring their attitudes and practices relating to infant feeding education. We transcribed and analyzed the interviews using the constant comparative method through the lens of the Theory of Planned Behavior by examining the participants’ attitudes, subjective norms, and perceived behavioral controls.

**Results:**

Teachers had predominantly positive attitudes towards the inclusion of breastfeeding education in high school parenting, child development, and nutrition courses, citing the need to normalize breastfeeding and support students’ ability to make informed choices when they become parents. Teachers’ subjective norms included concerns about parents’ and administrators’ views on the appropriateness of the content and apprehension about negative student responses. Perceived behavioral controls included student maturity, teachers’ own experiences and comfort with infant feeding, and the view that curriculum guidelines limit content selection.

**Conclusions:**

The results of this study can be used in the development and implementation of secondary school education programs that increase knowledge about infant feeding and positive attitudes towards breastfeeding for all members of the community. Teachers’ concerns need to be addressed in the implementation of these programs.

## Background

Breastfeeding rates have improved dramatically in the United States over the last four decades [1], and as of 2018, 83.2% of mothers initiated breastfeeding [2]. However, breastfeeding exclusivity is only 46.9% at three months and 24.9% at six months [2], indicating a need for increased breastfeeding education, promotion, and support. Documents from the U.S. Department of Health and Human Services (DHHS) [3], the WHO [4], and the UNICEF UK Baby Friendly Initiative (BFI) [5] outline strategies to increase breastfeeding initiation and duration. These documents recommend education programs aimed at increasing families’ and health care providers’ knowledge and skills regarding breastfeeding, which can promote positive attitudes toward the practice. The WHO [4] and the UNICEF UK BFI [5] recommend that schools include breastfeeding education for both male and female students to prepare them to make informed choices about infant feeding when they become parents. Additionally, many children have considered how they will feed their infants when they become parents and are interested in learning about breastfeeding [6]. Educational interventions with elementary and secondary school students may increase their knowledge about infant feeding and positively affect their attitudes toward breastfeeding [7]. Breastfeeding education in schools can provide students from various socioeconomic and cultural backgrounds with information about infant feeding early in their decision-making process.

There is limited research on teachers’ knowledge, experiences, and views on infant feeding education in the classroom [6, 8]. While most of the existing research on breastfeeding education in schools explores students’ knowledge of and attitudes towards breastfeeding [6, 7], there has been minimal research on teachers’ beliefs about including breastfeeding education in schools [9, 10]. Of the research done, the majority of teachers agree that information about the benefits of breastfeeding should be incorporated into the secondary school curriculum, particularly in science, health, and family sciences courses [8–10]. However, most teachers are not teaching about breastfeeding in their classes [8, 10]. Some indicate that they face barriers including time constraints and that the curriculum does not require breastfeeding content, others are concerned that breastfeeding education is inappropriate for mixed-gender classes and that it may encourage teen pregnancy [10]. The purpose of this study was an exploration of North Carolina (NC) Family and Consumer Sciences (FCS) teacher infant feeding education practices in their classrooms and their beliefs about breastfeeding education in secondary schools. FCS courses at the secondary school level include human development, nutrition and food science, hospitality, textiles and apparel, finance, and interior design [11]. As there are differences in school-age classification internationally, for this paper we use the term secondary school for United States middle and high school levels (approximate ages 12–18 years).

### Theoretical model

The Theory of Planned Behavior (TPB) 12 has been used to explain teachers’ implementation of curricula [12–15] and prioritization of teaching goals; therefore, it was identified as a suitable theoretical framework by which to analyze and organize the emerging themes from the interviews. According to the TPB, teachers’ views and attitudes regarding teaching about breastfeeding guide their intention to teach about breastfeeding within their classes. This framework outlines the individual motivators that guide a person’s actions, in this case teaching about breastfeeding in FCS classes. According to Ajzen [16], three factors guide a person’s intention to perform a behavior: 1) attitude toward the behavior, 2) subjective norms, and 3) perceived behavioral control. These factors combine to form the intention to perform the behavior. Depending on the persons’ realized control of the behavior, their intention guides the performance of the behavior.

## Methods

### Participants and recruitment

**Fig. 1:**
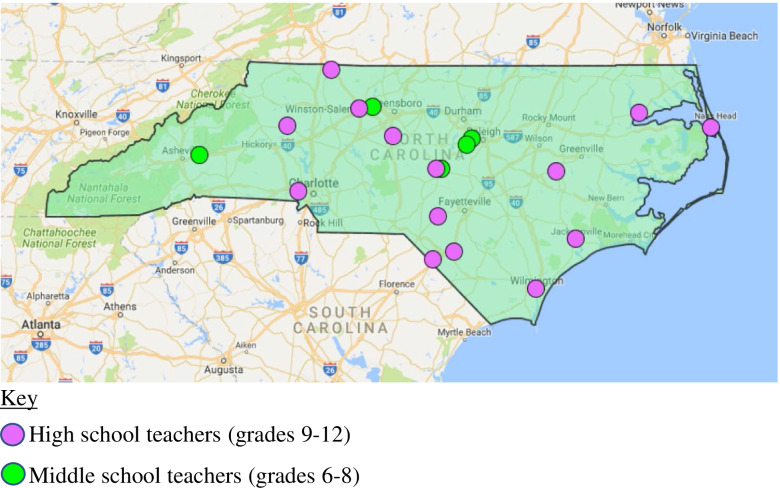
Map of Participants’ Teaching Location in North Carolina

The research team gathered teacher email addresses for study recruitment from school websites through a systematic county-by-county approach. We recruited current secondary school FCS teachers from NC public schools through an email invitation sent to teachers’ emails, college level FCS teacher education program coordinators (for distribution to former student teachers), and the NC Department of Public Instruction FCS listserv. We used a purposive sample (*N* = 19) [17] to ensure diverse participants; selecting participants from a variety of geographic settings, grade levels and FCS classes taught, and demographics based on their initial interest survey response. All participants were female and were diverse in terms of age, race/ethnicity, years teaching FCS, and education level (Table 1). They also represented a range of subjects and grade levels taught within FCS. The primary researcher determined sample size by saturation, which was confirmed by peer review, whereby no new themes emerged during analysis after the 14^th^ interview; we conducted additional interviews to verify saturation and ensure that middle school (grades 6–8, ages 11–14 years) teachers were represented in the sample [17]. The 19 participants interviewed were located throughout NC, as shown in the map of participants’ teaching location in NC (Fig. 1). NC State University’s Institutional Review Board granted this study [protocol number 6636] exempt from a full board review on the 23rd February, 2016, because it was considered to have minimal risk to participants.

**Table 1:** Demographic Descriptive Statistics for FCS Teacher Interviewed (*N* = 19)

Demographics	N	%
Gender		
Female	19	100
Male	0	0
Education		
Bachelors	11	58
Masters	8	42
Current teaching assignment		
Middle school	5	26
High school	14	74
Age (years)		
20 to 39	8	42
40 and older	11	58
Years teaching FCS		
0 to 9	10	53
10 +	9	47
Ethnicity		
Caucasian	12	63
African American	4	21
Other	3	16

### Procedure

The primary researcher used individual semi-structured telephone interviews to explore teachers’ infant feeding education practices and their attitudes towards breastfeeding education in the secondary school classroom (Table 2). In addition to primary interview questions, we used probes to facilitate discussion and clarify information [17]. The primary researcher conducted and recorded individual telephone interviews between March 2016 and May 2017. Participants also completed a demographic questionnaire before the interview. The researcher sent the consent forms by email before the scheduled telephone interview, and participants provided verbal informed consent and were enrolled in the study before beginning the interview; they received a $25 gift card upon completion of the interview.

**Table 2:** Primary interview questions for secondary school FCS teachers

Infant Feeding Education Practices
1. Tell me about classes you teach that include the topic of taking care of babies and children? Are there any classes you’ve taught in the past that covered this topic? 2. Tell me about classes that you teach or have taught that include the topic of feeding babies and young children 3. Please walk me through a typical unit or class you teach about infant and young child feeding a. Do you ever discuss infant feeding, including formula feeding and breastfeeding with the students in your classroom? b. Can you describe what topics you cover? 4. How do you decide what content to cover in your units on infant care and feeding? 5. What materials do you use to teach lessons about infant care and feeding? 6. Where do you get your curriculum, lessons, textbooks and/or supplemental materials? 7. How much time do you spend teaching about infant feeding? 8. How do your students respond to the lessons about infant feeding? 9. Compared to other topics you teach, how confident are you in your ability to teach infant feeding?
**Breastfeeding Education Attitudes**
1. What are your thoughts about including content about breastfeeding in middle and high school programs of study? 2. In what subject/s would breastfeeding content best fit into the curriculum? 3. What grade level/s would be best to teach about breastfeeding? 4. What are some reasons breastfeeding should be taught in the school setting? 5. What are some reasons breastfeeding should not be taught in the school setting? 6. What challenges or barriers do you/would you face including breastfeeding education in your classroom? What challenges or barriers do others face? 7. What information would you like or need to teach about breastfeeding? 8. Are there any resources or training that you would like or need to teach about breastfeeding?

### Data analysis

Trained research assistants transcribed audio recordings verbatim, the primary researcher compared each transcript with the recording for accuracy, and we imported them into Dedoose [18]. The primary researcher used the constant comparative method for data analysis, cycling between data collection and preliminary analysis in rounds to allow the analysis to guide subsequent interviews [17]. After initial coding, the primary researcher organized the codes regarding teachers’ attitudes on breastfeeding education into categories and sorted them into themes within the context of the three motivators of the TPB: attitude toward the behavior, subjective norms, and perceived behavioral control.

We established trustworthiness through several methods during data collection and analysis:

- Triangulation using a range of documents mentioned by participants [19]. The primary researcher used textbooks, NC curriculum blueprints, and other curriculum materials to verify details provided during the interviews.
- Member checks at the end of each interview allowed the interviewees to provide clarification, expansion, or correction of statements [19, 20].
- An internal committee (the primary researcher’s dissertation committee, comprised of tenured faculty who are experts in qualitative research and human lactation) and blinded external peers not involved with the research (tenured faculty at R1 institutions and experts in qualitative research in education) conducted peer debriefing to counteract potential bias of the primary researcher [21]. Peers examined and provided feedback on the methodology, interpretation, and analysis of the data after every three interviews were analyzed. Feedback was incorporated in to subsequent interviews and analysis [22].

The primary researcher established the dependability of the study by using an audit trail, documenting methodological decisions, and writing reflective memos before and after each interview and at other times when reflection occurred [19]. These documents were used as part of the internal and external peer review process, as a mechanism of trustworthiness.

## Results

**Fig. 2:**
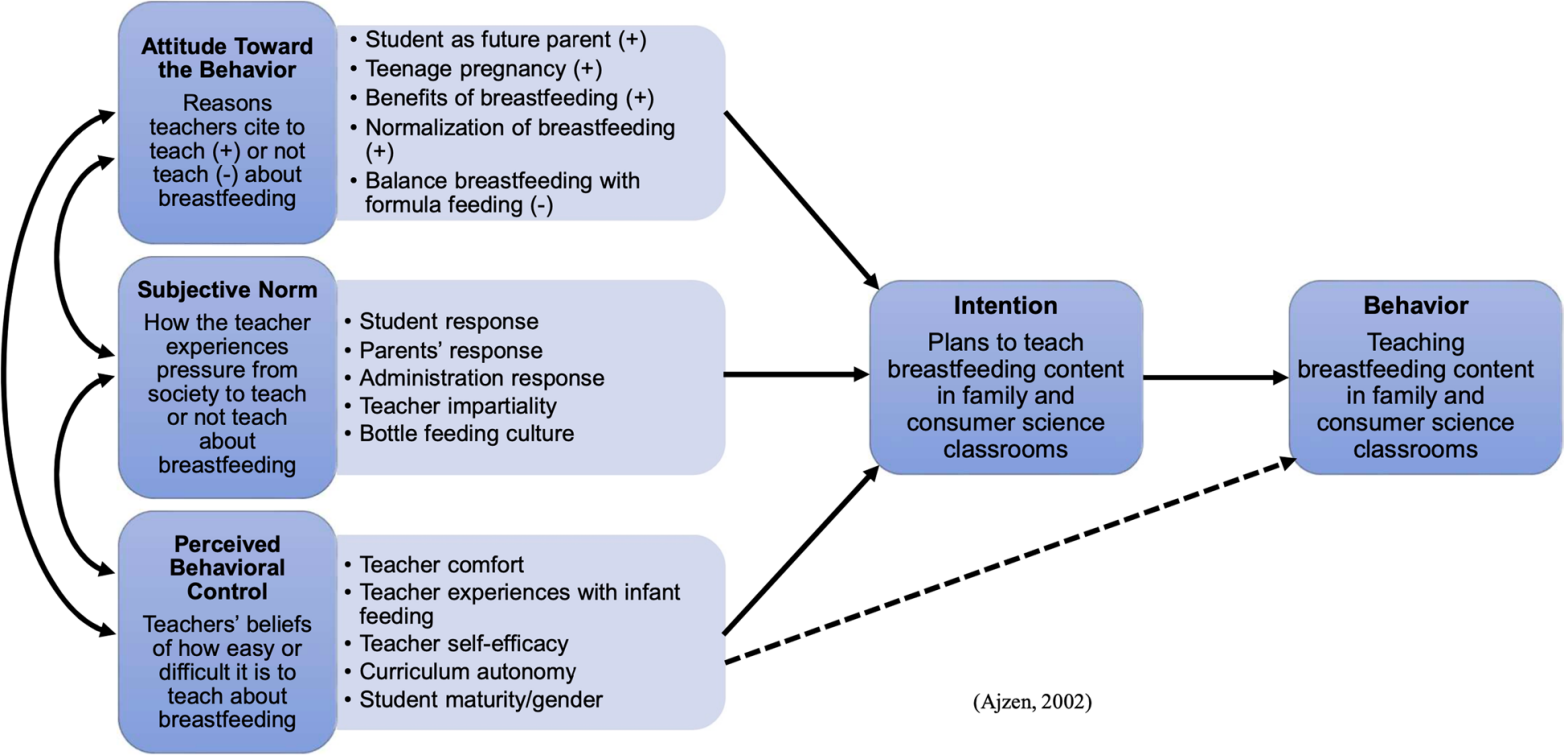
Thematic Representation of NC Family and Consumer Science Teachers Attitudes Towards Breastfeeding Education in Secondary School Classrooms in the Context of the Theory of Planned Behavior

As discussed in the following sections organized by theme, the following beliefs guide participants’ intention to teach about breastfeeding in their classrooms: 1) their attitudes towards teaching about breastfeeding, 2) their subjective norms on breastfeeding education, and 3) their perception of factors that control what and how they teach in their classes. Figure 2 shows a thematic representation of NC FCS teachers’ beliefs about breastfeeding education in secondary school classrooms within the context of the TPB (attitudes, subjective norms, perceived behavioral control).

### Attitude toward the behavior

The TPB suggests that a person’s attitude towards a behavior is determined by their belief about the consequences of the behavior (behavioral beliefs), which create their favorable or unfavorable attitude towards the behavior [16]. Therefore, teachers’ views and attitudes regarding teaching about breastfeeding guide their intention to teach about breastfeeding in their classrooms. The participants cited many reasons to teach about breastfeeding related to positive student outcomes and had limited negative attitudes towards teaching about breastfeeding in their classes. The themes presented by teachers as reasons to teach about breastfeeding were: student as future parent, teenage pregnancy, benefits of breastfeeding, and normalization of breastfeeding. Some teachers felt that they needed to balance educating about breastfeeding with formula feeding for those who are not able to or choose not to breastfeed.

#### Student as future parent

There was an emphasis that many students (both male and female) will become parents in the future and would benefit from complete information about the feeding choices for their infants.

*I definitely think it should be [taught], especially in the high school setting, because most of these kids are gonna be parents at some point - if they’re not parents already - and I think that it would be a huge disadvantage to not teach our children about the benefits of breastfeeding.* (Teacher #13, HS)

#### Teenage pregnancy

In addition to the future role as a parent, several teachers mentioned that they have students who are already parents or will become parents as teenagers. They cited that the chance of teen pregnancy was a reason to teach students about breastfeeding as early as middle school, which is the age at which some students become sexually active and therefore may become pregnant.

*However, we are having a lot of younger [students], entering high school in the ninth grade becoming parents, so I think it needs to be taught as soon as possible or earlier.* (Teacher #7, HS)

*If we’re teaching kids where babies come from and they’re having sex, you can’t not teach about breastfeeding. That’s one hundred percent, you better be doing that. That has got to be in the curriculum.* (Teacher #8, HS)

#### Benefits of breastfeeding

More than half of the teachers mentioned the benefits of breastfeeding as justification for including the information in the classroom. They described the health benefits for mothers (weight loss and return of uterus to pre-pregnancy size) and children (nutrient availability, immune benefits, obesity prevention, intellectual development, prevention of sudden infant death syndrome) as well as the financial benefits for the family.

*When the mother is producing exactly what the child needs and it’s free, and that is one of the points that I make to them that it’s…a money saver.* (Teacher #3, HS)

*Yeah, I think it’s important because a baby gets so many nutrients through breast milk that they cannot get through formula, so it’s important to teach moms about those needs and how important they are and how important those antibodies are for protection from sicknesses and things like that.* (Teacher #2, HS)

#### Normalization of breastfeeding

Several teachers recognized that breastfeeding education in schools has the capacity to normalize breastfeeding in society, both as a way that parents feed babies and to make breastfeeding in public more socially acceptable.

*You can almost see the little light bulbs turn on their heads when we are talking about breastfeeding because they just never thought of it in that way because they don’t have someone in their life that they have seen breastfeeding. All they know is formula feeding and, it’s sad that their limit of their education stops at a bottle, plain and simple.* (Teacher #3, HS)

*Especially now because you do hear about women who breastfeed in public and they get comments made to them and it’s just somehow not socially acceptable to do it in public…I think it is good for them to know that it is just a normal part of life.* (Teacher #10, HS)

#### Balance breastfeeding with formula feeding

Two teachers expressed concerns that promoting the health benefits of breastfeeding undermines families who formula feed. They described the need to balance breastfeeding content with formula feeding for mothers who cannot or choose not to breastfeed.

*I just say, don’t put the stigma on people that don’t breastfeed, that they’re not any less of a parent because they don’t do it…And that there’s people that struggled and they tend to feel that they’re not as good of parent because they can’t breastfeed, and I just don’t think that is something that any mom should have to feel because if you can’t, you can’t*. (Teacher #10, HS)

### Subjective norms

Teachers indicated that the normative beliefs of other people are an important determinant of their behavior. Their subjective norms about breastfeeding education included how students respond to the material, their perception of parent and administrator views on including this topic in the classroom, how teachers should present information impartially, and how bottle-feeding is a cultural norm in our society.

#### Student response

Student responses to breastfeeding content were a consideration for teachers when incorporating this information in their classes. Teachers described their students’ responses range from fascination to disgust. Some students were initially embarrassed or uncomfortable about the anatomy of the breast, but many were interested in the benefits of breastfeeding and about feeding infants in general.

*Now, they tend to be getting kind of squirrely if there is any breast shown at all in the videos, which is very discrete, but they know that it’s breastfeeding, so most kids are not comfortable with that part of it.* (Teacher #3, HS)

One teacher explained that students who were already parents expressed their interest in breastfeeding by asking questions and said they would try breastfeeding with their children because of the benefits.

*Most of them are really interested in breastfeeding and the parents in class, they ask a lot questions, and I feel like a lot of them, after teaching that lesson, are persuaded to try it. Like I hear them say, “I’m going to do breastfeeding, I’m going to try it, I’m going to do it. You know it’s free first of all, it’s good for the baby, it’s good for me. I promise you I’m going to do my best to try.”* (Teacher #7, HS)

#### Parents

Teachers felt that parents would want the schools to inform them about the education of their children regarding their bodies and sexuality. Several teachers had concerns that parents would view teaching about breastfeeding as encouraging sexual activity and teenage pregnancy. To inform parents, a couple of teachers use permission slips for content that shows the naked human body or discusses information related to sexual activity.

*Parents sometimes have this idea in their head, I feel, that the minute you start talking about anything to do with sex or babies or whatever, that you’re trying to encourage them to have babies, which is not what it is. It’s really the opposite, because I believe you need to be ready to have a child, to take care of that child.* (Teacher #12, HS)

*And I ask, I have to get permission, and I ask ahead and get the parents to sign off on that… I just tell them that there will be a breast, an actual woman’s breast in the video.* (Teacher #9, HS)

#### Administrators

Two teachers described that school administration may not approve of including breastfeeding content in their classrooms. One teacher talked about the feedback she received from the school system on buying breastfeeding clips for the baby simulators used in parenting courses. She convinced them to purchase the clips and now uses them in her classes but has to provide students the option to bottle feed when they take the baby simulators home.

*Even when I got the plastic babies and things, the school system didn’t want to buy the little monitors they clip on their shirt to simulate breastfeeding; they just wanted to do the bottles and I said, “no, no, no, no, we really need this. If I’ve got to write a grant or I need to do something, we need to show children that that is the best way to go”…they allowed me to get two of them for the ten babies, but I still have to offer the children the option of just doing the bottle. They were supportive; I wish they had been more supportive.* (Teacher #8, HS)

#### Teacher impartiality

Teachers verbalized that they should be teaching information based on evidence, not based on their personal views or opinions on a topic. As a result, they would not influence the students’ views but present lessons from a factual basis to promote student choices based on evidence.

*Saying okay let’s have everyone go to breastfeeding only, you can’t do that, you can’t voice your opinion. We’re teachers, your opinion is to be quiet and teach and allow the child or the student or whoever to make the choices for themselves.* (Teacher #11, HS)

#### Bottle feeding culture

When asked about the infant feeding content they cover in their classes, some teachers responded primarily from a bottle-feeding perspective while others talked primarily about breastfeeding until prompted for the other content area.

*I teach Foods and Nutrition and we have a section there on making and preparing meals, nutritious meals for children. Infant feeding, you know what age you should start feeding them foods and we talk about the formula, of course they say “milk” but, you know, formula.* (Teacher #9, HS)

Teachers’ social norms of infant feeding methods appeared as they described the detail of their lessons. One teacher talked about feeding in a way that showed normalization of formula feeding. When asked about infant feeding, first she responded with information about bottle feeding, followed by detail about the kits that she has for bottle feeding and the parts of bottle feeding she emphasizes in her classroom.

*Early Childhood 1 does have a section where students have to demonstrate how to feed a baby, and it’s a little kit and it has props and stuff. I actually made it into a little kit where you give them the items the need like the bottle, the bib, everything they’re going to need to do the procedure.* (Teacher #5, HS)

### Perceived behavior control

A person’s perception of behavioral control can affect both their intention to perform the behavior and the actual performance of the behavior [16]. Teachers’ beliefs of how easy or difficult it is to teach about breastfeeding included intrinsic factors, such as their comfort with the information and their self-efficacy teaching about infant feeding, which they related to their education and experiences with infant feeding. The extrinsic factors that control how they teach infant feeding content included curriculum autonomy, student maturity, and student gender.

#### Teacher comfort

Many of the teachers interviewed were comfortable teaching about the human body, and they related breastfeeding to other topics that they teach within the Parenting and Child Development course such as pregnancy, birth, and sexually transmitted diseases. They described that the addition of breasts and breastfeeding was a comfortable topic despite teenagers’ embarrassment or discomfort with topics related to bodies and sexual development. Other teachers felt that teaching about these topics could be awkward.

*I can imagine a young woman in FCS that has not had children might feel that there’s a barrier for her, but being a mother who has breastfeed herself there were, there has been none for me and I don’t see any in the future to be honest.* (Teacher #3, HS)

*I think that sometimes teaching, especially for me, teaching about childbirth and conception and infancy, sometimes that can be uncomfortable for teachers and for students, just because it can sometimes. It can be considered a private issue.* (Teacher #1, HS)

#### Teacher experiences

The teachers’ personal experiences with infant feeding through their education, childcare experience and/or parenting shape the way they teach their students about infant feeding in their classrooms. The teachers who are parents talked about their own experiences feeding their children (both positive and negative) and tied those experiences into their classrooms. Those without children talked about the challenges of teaching parenting and infant feeding before they have children of their own.

*I think the one disadvantage that I have is that just due to our careers and even some medical issues, I never was a parent myself and so I never had the opportunity to breastfed myself.* (Teacher #15, MS)

One teacher talked about her positive breastfeeding experience but acknowledged that not everyone has this experience. She tries to give her students a realistic view of infant feeding.

*I’m a big advocate for breastfeeding, I had a wonderful experience with it. I know that everybody does not, so I try to also talk about a lot of the issues and why we think people get discouraged with it, and I’m very honest that it is painful and starting out it’s very hard but the benefits outweigh it...Really, it’s just helped me be honest.* (Teacher #7, HS)

#### Teacher self-efficacy

Teachers’ described that their education combined with personal experiences raising their own children and with childcare guide their selection of materials and create self-efficacy for teaching infant feeding content.

*I feel pretty confident because I’ve taught it and I’ve also experienced it as a mother. So, I think it’s helpful to have the personal experience, and I feel like I can answer the questions if need be, and if I don’t know the answers to the questions I can always go to a lactation specialist*. (Teacher #17, HS)

#### Curriculum autonomy

Teachers’ views on the amount of autonomy they have in choosing and presenting content varied, with some stating that the content they present is completely guided and controlled by the state standards and testing.

*Every school in North Carolina has the same curriculum put out by the state of North Carolina, and if you don’t follow those guidelines then your scores on the end of grade test, which in our case in called the CTE test … then they do not do well unless you follow the curriculum that is provided by the state*. (Teacher #11, HS)

Conversely, other teachers felt that they have more flexibility in the exact content that they teach in their classes. This teacher described how the state curriculum does not include much information on breastfeeding, so she felt that she could add relevant content as long as she could justify it within the infant feeding objectives.

*So, they give me standards and objectives that I have to cover, but personally, I feel like they are really broad, so I’m kind of wide open to teach whatever I want as long as it covers those standards.* (Teacher #1, HS)

#### Student maturity/gender

Teachers described that, as students progress from middle to high school, their cognitive development allows them to view life from outside their own individual needs and perspectives; therefore, they are better able to understand, synthesize, and apply information about breastfeeding.

*The maturity level, the ability to think outside of their own little bubble because a lot of them just cannot do that yet, they can’t think about caring for someone else because they can’t even care for themselves. And usually tenth grade, they are getting a little bit more independent, they’re starting to drive, so I think that helps strengthen their maturity level.* (Teacher #2, HS)

*I definitely think that in middle school I would focus on the babysitting aspect of babies receive their nutrition differently, I think in high school, depending on how you look at it, there are more and more teenagers who have babies of their own and I think that, and this is purely my opinion, I think they need to understand the benefits of breastfeeding verses bottle feeding. And by bottle feeding I’m talking about formula because I know there are lots of babies that are fed breastmilk through a bottle.* (Teacher #20, MS)

Some teachers viewed mixed gender classes as a barrier to incorporating breastfeeding content. This teacher described that female students view breastfeeding as personal content which makes them uncomfortable when she teaches it when male students are present.

*And I know we try to promote gender equality and all of that but it’s hard to get a teenager girl to sit there and talk about breastfeeding when there’s boys sitting there too. Like sometimes, they’re a little bit more resistant to ask you questions or actually talk to you more about things.* (Teacher #2, HS)

## Discussion

The teachers in this study described three motivators within the framework of the TPB that provide insight into their intention to teach about breastfeeding in their classes as well as their inclusion of breastfeeding content. Teachers’ personal attitudes (attitude toward the behavior), how they feel society views teaching about breastfeeding education in schools (subjective norms), and their perception of factors that control their behavior (perceived behavioral control) guides their inclusion of breastfeeding education in secondary school classrooms [16].

One motivator was the teachers’ personal attitudes towards the behavior. Similar to other research with teachers [9, 10], they presented generally positive attitudes towards teaching about breastfeeding in schools citing the need for students to be informed when they become parents. Teachers understood that incorporating information on the benefits of breastfeeding in the classroom has the potential to normalize breastfeeding in society. However, the view that they should present formula feeding as equal is a barrier to accurate infant feeding information because the risks of formula feeding for mothers and children are well established [3, 23, 24]. It is important to provide teachers with a selection of lessons that provide varying depth of content while acknowledging teacher concerns about providing impartial information to students through evidence-based materials. Future curriculum planning and implementation should include content about the benefits of breastfeeding and the risks of formula feeding (with continued coverage of safe formula preparation) [3, 25, 26] while remaining sensitive to the choices that students and their friends or families may have made to formula feed.

Another motivator was how teachers’ subjective norms shape their decisions about whether and how to teach information about breastfeeding in their classes. They described that student responses to lesson content about breastfeeding was varied, both positive and negative. This finding supports research with secondary school students showing similar mixed attitudes towards breastfeeding with both positive, neutral, and negative attitudes previously described [6]. Generally, teachers felt comfortable handling negative student responses due to their experience teaching other concepts related to the human body. To address teacher comfort with the material, lessons can target classes where information about the human body is already included in the curriculum such as FCS, health, or science. Additionally, some teachers voiced apprehension that parents and administrators would view teaching about breastfeeding as promoting teen pregnancy. This expressed apprehension confirms Spear’s work [10] in which teachers were concerned that breastfeeding is a sensitive topic that can be linked to teenage pregnancy [10]. Schools could address teachers’ concerns about parents’ sexual perceptions of breasts and breastfeeding by implementing permission letters for this content; however, this may perpetuate the idea that breastfeeding is a sexual topic.

The final motivator was the teachers’ perceptions of factors that control their behavior (both intrinsic and extrinsic). Intrinsic factors included teachers’ knowledge, experience, and comfort with the content. Prior research outside of the United States indicates that teachers are familiar with the basic benefits of breastfeeding, but have misconceptions regarding detailed infant feeding recommendations, which could contribute to their lack of self-efficacy [6]. While prior work indicates gaps in teacher knowledge internationally, there is currently a dearth of research on teachers’ knowledge about breastfeeding in the United States. Our research indicates that teachers’ personal experiences with babies and children shape the way they teach their students about infant feeding, demonstrated in their attitudes towards including breastfeeding information in their classes and the content they teach. Teachers who had experience with childcare or as parents described comfort and self-efficacy in presenting information about infant feeding to their students. Teachers who do not have personal experience with breastfeeding and/or infant feeding will need professional development opportunities to implement new content successfully.

Teachers’ extrinsic behavioral control perceptions centered on their autonomy within the FCS curriculum. Generally, teachers were open to including breastfeeding content in FCS courses that cover infant development or nutrition during the life cycle but voiced obstacles that confirm earlier research in which teachers reported they have limited time to include breastfeeding education in their courses, particularly when it is not part of the required curriculum [10]. How teachers interpret the infant feeding objectives in course guidelines varied, as did their views on their autonomy within the curriculum. Most of the teachers interviewed felt that they could add material to the state curriculum as long as it fits the needs of their students and stays within the outline of the course provided by the Department of Public Instruction. Curriculum developers should design lessons on infant feeding in collaboration with national and state level education departments to meet curriculum goals thereby reducing teachers’ perceptions that the curriculum does not cover the content in detail or at all. Including more specific breastfeeding content in the mandated curriculum would make it easier to incorporate in the classroom, especially for teachers who are not parents or are less familiar with evidence-based infant feeding recommendations. Uptake of breastfeeding curriculum materials could be facilitated through the use of existing curricula [27, 28], as well as collaboration with breastfeeding organizations such as the United States Breastfeeding Coalition, local breastfeeding coalitions, public health departments, and extension programs.

This paper is the first to explore acceptability of breastfeeding education for different ages of secondary school students. Teachers echoed research indicating that incorporating breastfeeding education in high schools is more acceptable than in middle school [10, 29–31]. They described hesitation about the developmental readiness of middle school students for specific content about breastfeeding. To address teachers' concerns about student maturity, courses at the high school level such as parenting and child development, nutrition, and health are the ideal location for more detailed curriculum materials on infant feeding and breastfeeding. While most teachers were more open to including this content at the high school level, some felt it should be included in middle school to begin to normalize the conversation about breastfeeding and in case students become pregnant during early adolescence. Based on teacher hesitation to teach breastfeeding in FCS curriculum due to their assessment of the maturity of middle school students when compared to high school students, content could be integrated into the middle school curriculum as an overview of infant feeding (within the context of childcare and babysitting which are already covered in many middle school FCS classes) and expanded upon at the high school level in courses like Parenting and Child Development as well as Nutrition.

## Conclusions

This qualitative study contributes to research that guides the development and implementation of secondary school education programs that increase knowledge about infant feeding and positive attitudes towards breastfeeding for all members of the community. The teachers in this study indicated positive attitudes about including breastfeeding education in FCS classes, particularly at the high school level; however, they had concerns about parent and administrators’ views of the content and curriculum limitations. One limitation of this study is that the findings may not represent the views of all NC FCS teachers due to selection bias. Study recruitment used wording that emphasized infant feeding rather than breastfeeding to reduce the likelihood of recruiting only participants with strong positive or negative views about breastfeeding. This study is the first to gather robust qualitative data about teachers’ attitudes towards breastfeeding education in schools and begins to address the gap between research and practice. Addressing teacher concerns will facilitate the inclusion of more breastfeeding information in schools. Furthermore, understanding teachers’ beliefs about breastfeeding education within the context of the TPB can allow for targeted curricula and professional development for educators that address the motivators that guide their actions.

Future research could focus on 1) mixed methods studies building on the TPB described in this study to explore regional beliefs about breastfeeding education in school that may affect teacher motivators, 2) work with other stakeholders such as students, parents, and administrators regarding their views of breastfeeding education in schools to confirm teachers’ perceptions that this content is controversial, and 3) curriculum design and pilot testing that addresses teacher willingness and ability to uptake materials into their classrooms both at the middle and high school level.

## Data Availability

Data sharing is not applicable to this article as no datasets were generated or analyzed during the current study. Interview transcripts are not allowed to be shared per IRB.

## Abbreviations

FCS: Family and consumer sciences
NC: North carolina
TPB: Theory of planned behavior
BFI: UNICEF UK Baby friendly initiative
DHHS: U.S. Department of health and human services
WHO: World health organization
